# Human clinical trial using diagnostic ultrasound and microbubbles to enhance neoadjuvant chemotherapy in HER2- negative breast cancer

**DOI:** 10.3389/fonc.2022.992774

**Published:** 2022-10-20

**Authors:** Biqiang Zhou, Qingshu Lian, Chunchun Jin, Jianghao Lu, Lifeng Xu, Xuehao Gong, Peng Zhou

**Affiliations:** ^1^ Department of Geriatric & Spinal Pain Multi-Department Treatment, The First Affiliated Hospital of Shenzhen University, Shenzhen Second People’s Hospital, Shenzhen, China; ^2^ Department of Ultrasound, The First Affiliated Hospital of Shenzhen University, Shenzhen Second People’s Hospital, Shenzhen, China

**Keywords:** ultrasound, microbubbles, HER2-negative breast cancer, neoadjuvant chemotherapy, cavitation

## Abstract

**Background:**

*In vivo* and *in vitro* experiments have demonstrated that diagnostic ultrasound combined with microbubbles (USMB) can enhance tumor chemotherapy, but few clinical studies have explored the effect of USMB in human HER2-negative breast cancer. We aimed to compare USMB combined with neoadjuvant chemotherapy (NAC) with NAC alone in the treatment of human HER2-negative breast cancer.

**Methods:**

Patients (n=10) enrolled in the study were treated with TAC (taxane – (docetaxel), anthracycline – (epirubicin or doxorubicin liposomes), and cyclophosphamide) and ultrasound using a commercial clinical ultrasound scanner for 20 min after each chemotherapy session, followed by intermittent injections of SonoVue^®^ to induce sonoporation and enhance therapeutic efficacy. Contrast-enhanced ultrasound (CEUS) was used to record tumor perfusion before and after ultrasound treatment.

**Results:**

After completion of chemotherapy, the maximum tumor diameter of patients in the combined treatment group (n=10) was significantly smaller than that in the control group (n=16) (*p*=0.017). Although the combined treatment group had higher overall response and clinical benefit rates than those in the control group, there was no statistically significant difference in RECIST between the combined treatment group and the control groups (*p*=0.590). More patients in the combination therapy group achieved pathologic complete response than in the control group (*p*=0.014). For combined treatment, CEUS revealed that the peak intensity, mean transit time, and area under the curve were higher after treatment than before treatment (*p*<0.001, *p*<0.001, *p*=0.003, respectively). Combined therapy did not cause additional toxicity or increase side effects.

**Conclusion:**

USMB and chemotherapy can be combined in a clinical setting using commercially available equipment, without additional toxicity, and may improve the efficacy of NAC in HER2-negative breast cancer.

## Introduction

Breast cancer exhibits the highest incidence of malignancy, threatening the health of women worldwide (1). Neoadjuvant chemotherapy (NAC) is an important treatment option for locally advanced breast cancers. NAC involves systemic chemotherapy before surgery to kill tumor cells, shrink the tumor volume, reduce the breast cancer clinical stage, provide inoperable patients with opportunity for surgery, or reduce the surgical excision area and improve the possibility of breast-conserving surgery, which significantly enhances the quality of life in patients (2). The long-term survival of patients who achieve pathologic complete response (pCR) after NAC is significantly improved. In clinical studies, the pCR rate is considered to be the endpoint of the long-term efficacy of NAC, but there are significant differences in pCR rates among different subtypes (3, 4). According to the study, the pCR rate of the HER2 type was higher than those of the triple-negative and luminal B types. In the luminal B type, the pCR rate of the HER2-negative type was lower than those of the HR+ and HER2+ types (5). Due to the lack of effective targeted drugs for HER2-negative breast cancer, particularly triple-negative breast cancer, the prognosis is generally considered poor and has a higher risk of recurrence or metastasis (6). Therefore, improving the sensitivity of HER2-negative breast cancer to NAC drugs is an urgent issue that must be addressed in the current clinical treatment of breast cancer.

In recent years, ultrasound combined with microbubbles (USMB)-induced cavitation has been widely applied in medical research for the treatment of diseases. Cavitation refers to the biological effect induced by acoustic waves, which is mainly manifested as the formation of temporary and reversible pores on the celluar membranes and vessel walls to enhance transport of therapeutic agents across these natural barriers within the target area of irradiation (7), generally lasting for 1–4 h (8). It is generally believed that under the same conditions, the higher the ultrasonic intensity, the higher the concentration of cavitation nuclei and the more significant the cavitation effect. Under normal circumstances, the concentration of cavitation nuclei in the body fluids of organisms is very low, and the cavitation effect requires high-intensity ultrasonic irradiation. In addition, microbubbles can be used as artificial cavitation nuclei, enter the body intravenously, and accumulate in the target area, which can increase the local concentration of cavitation nuclei and reduce the threshold ultrasonic intensity. Therefore, the ultrasonic intensity of the cavitation effect is greatly reduced (only 0.5–2 W/cm^2^), within the scope of the sound intensity level in diagnostic ultrasound. Using only diagnostic ultrasound plus microbubbles can produce desirable cavitation (8–10). Many *in vitro* and *in vivo* studies have demonstrated that USMB is a viable technique for enhancing drug delivery and improving therapeutic efficacy (11–13).The experimental results of Gourevich and Chen et al. confirmed that USMB can increase the concentration of chemotherapy drugs in breast cancer cells and increase the killing effect of chemotherapy drugs on tumor cells (6, 14). Dimcevski et al. have shown that ultrasound combined with contrast microbubbles may improve the clinical efficacy of gemcitabine, prolong the quality of life, and extend survival in patients with pancreatic ductal adenocarcinoma (15).

Therefore, ultrasound-stimulated contrast agent microbubbles combined with chemotherapy is expected to become a novel, safe, and noninvasive treatment method. Therefore, this study aimed to apply diagnostic ultrasound combined with contrast agent microbubbles to patients with HER2-negative breast cancer receiving NAC. This study aimed to compare the clinical efficacy and safety of USMB combined with NAC with conventional NAC in the treatment of HER2-negative breast cancer.

## Materials and methods

### Subjects

From April 2019 to October 2020, we enrolled 10 patients with inoperable breast cancer at Shenzhen Second People’s Hospital. All patients were histologically confirmed to have locally advanced HER2-negative invasive breast carcinoma, including luminal B HER2-negative [HR+ (ER+ and/or PR+) and HER2 –] and triple-negative [ER –, PR –, and HER2 –]. NAC was performed at Shenzhen Second People’s Hospital; mastectomy was performed after completion of chemotherapy. Patients were required to meet the criteria for NAC at our hospital and possess no known intolerance to chemotherapeutic agents or SonoVue^®^ (Bracco Imaging S.P.A, Milan, Italy).

Data from breast cancer patients who received the same NAC at Shenzhen Second People’s Hospital from February 2019 to December 2020 were collected as the control group to compare the efficacy and safety of treatment. In terms of treatment, the only difference between the conventional NAC group and the ultrasound treatment (USMB + NAC) group was the addition of ultrasound and microbubbles after chemotherapy. This study was reviewed and approved by the Ethics Committee of our hospital.

### Chemotherapeutic plan

Two experienced oncologists who had performed chemotherapy were not included in the study. We used the standard recommended treatments for TAC: taxane (docetaxel), anthracycline (epirubicin or doxorubicin liposomes), and cyclophosphamide. The injection cycle was once every 3 weeks, with treatment suspended or dose adjusted according to the standard guidelines (16). Chemotherapy was continued as long as the treatment was effective, and surgery was performed after six rounds of chemotherapy.

### Ultrasound scanner configuration and microbubble dosage

**Figure 1:**
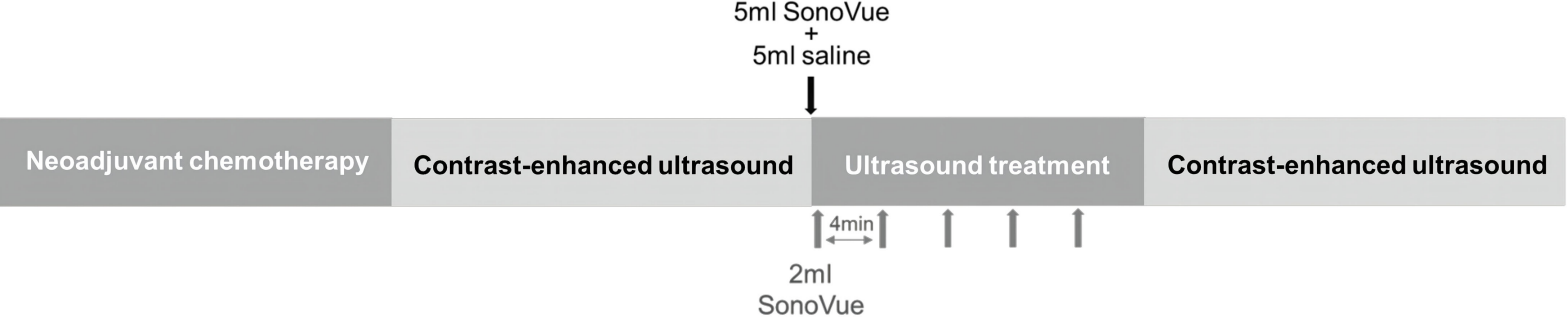
Flow chart of ultrasound therapy.

Patients in the USMB + NAC group were treated with ultrasound within 24 h after each infusion of drugs in each cycle of chemotherapy. Each session of ultrasound therapy lasted for 20 min. During ultrasound therapy, the patient lay supine on the examination couch. The displayed image of the VINNO70 ultrasonic therapeutic instrument (VINNO Technology Co., LTD., Suzhou, China) was used to set the required ultrasonic treatment mode and parameters (V-flash mode c, frequency: 4 MHz, pulse repetition frequency: 20 Hz, pulse time: 1.0 s, intermittent time: 5 s, treatment time: 1200 s, and mechanical index: 0.3–0.4). An X4-12L linear array probe was used to accurately locate the lesions required, and 5 mL SonoVue^®^ (Bracco Imaging S.P.A, Milan, Italy) ultrasound contrast agent microbubbles were injected through the cubital vein first, followed by 5 mL normal saline for internal circulation. SonoVue^®^ is a commercially available ultrasound contrast agent that contain 10^8^/mL of microbubbles with a mean diameter ranging from 2.0-4.0 μm after preparation. Subsequently, five consecutive slow intravenous injections of 2 mL SonoVue^®^ (Bracco Imaging S.P.A, Milan, Italy) were performed every 4 min. To enable a homogenous treatment of whole tumor, the probe was rotated by approximately 30° per min and kept the lesions be displayed at the center of the display screen throughout the entire procedure. GE LOGIQ E9 (GE, Milwaukee, USA) was used for conventional ultrasound examination and contrast-enhanced ultrasound (CEUS) immediately before and after the ultrasound treatment. During conventional ultrasound examination, a 6–15 MHz probe (ML6–15) was used to record the location, size, and morphological information of the breast cancer lesions. 3.0–9.0 MHz probe (9 L) was selected, and 5 mL contrast agent suspension was injected through the cubital vein followed by 5 mL normal saline in CEUS to record the blood perfusion of the lesion (**Figure 1**). The position and direction of the probe was marked on the patient during CEUS before treatment, so that the re-contrast after treatment could be observed under the same section as possible.

### Efficacy evaluation

Changes in the maximum tumor diameter in the USMB + NAC and NAC groups were recorded according to the Response Evaluation Criteria in Solid tumors (RECIST1.1). Therapeutic effects were divided into complete response (CR), partial response (PR), stable disease (SD), and progressive disease (PD). The overall response rate was defined as CR plus PR, and clinical benefit rate was defined as CR plus PR and SD lasting at least 4 weeks (17). The maximum diameter of tumor acquired using dynamic scan of conventional ultrasound was analyzed by a radiologist with over 10 years of experience in breast imaging. The reader did not know the patient whether given ultrasound treatment or not.

Pathological reactions after NAC were evaluated based on postoperative pathological results using the Miller–Payne (MP) system and residual cancer burden (RCB) system. The MP system compared the biopsy specimen before NAC with the surgical specimens after NAC, mainly evaluating the cell richness of the residual tumor of the breast primary tumor after NAC, which was divided into five grades. The RCB system evaluated the range of primary residual tumor of the breast (mm×mm), cell density of the residual tumor (%), proportion of carcinoma *in situ* (%), number of positive lymph nodes, and maximum diameter of residual metastatic lymph nodes (mm). After the calculation, the RCB index and corresponding RCB grade can be obtained, which can be divided into four grades. The MP-5 and RCB-0 grades suggest that pCR was achieved. When the two assessment systems do not agree regarding the recurrence risk of the same patient, the higher risk is taken (18).

### Tumor perfusion

**Figure 2:**
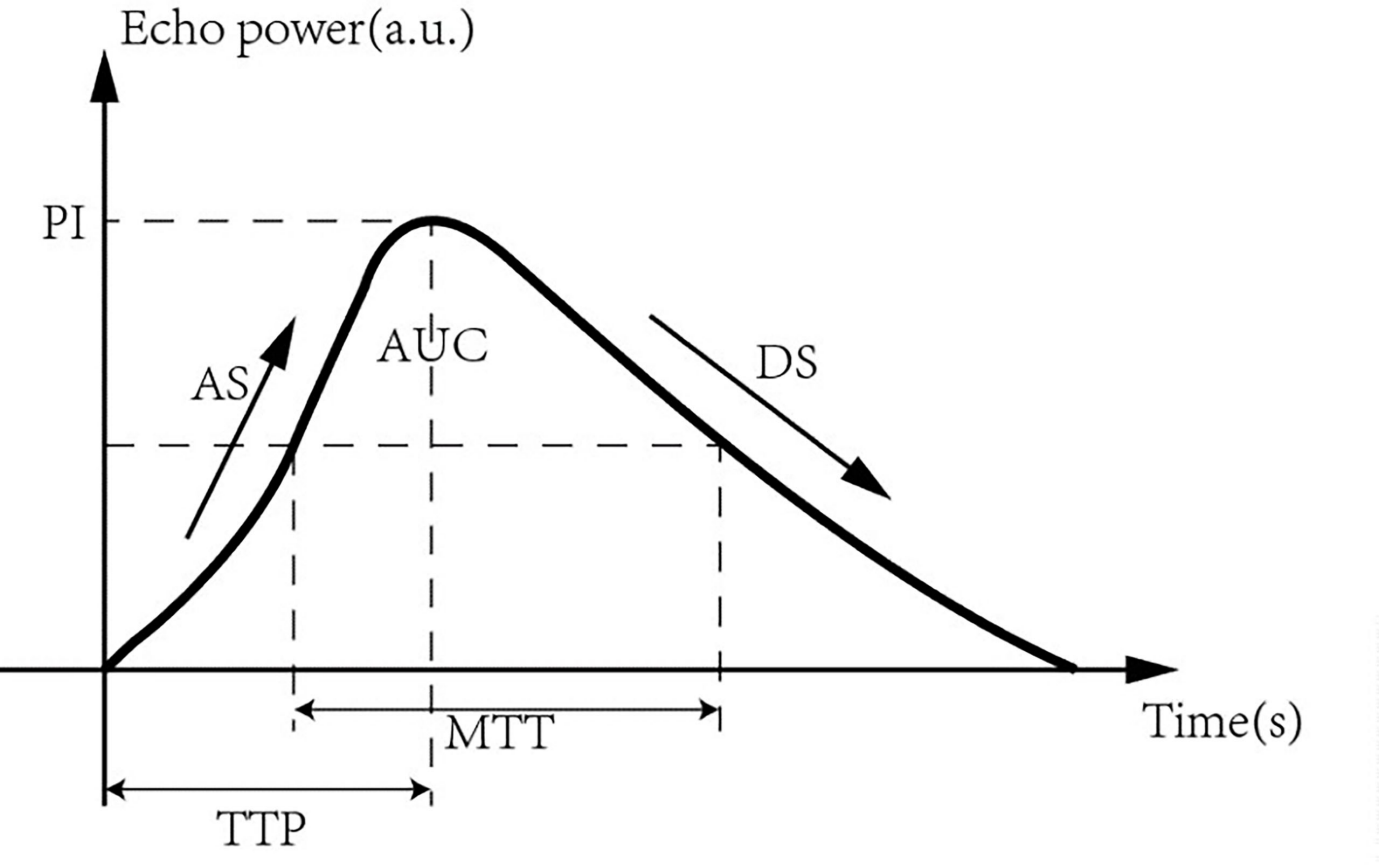
Time-intensity curve (TIC) of region of interest (ROI) generation in the lesion.

The region of interest (ROI) of the lesions was analyzed using the quantitative analysis software GE LOGIQ E9 (GE, Milwaukee, USA) ultrasound diagnostic instrument. Quantitative analysis was performed to compare parameters of the time-intensity curve (TIC) in the ROI before and after ultrasonic therapy, including peak intensity (PI), time to peak (TTP), ascending slope (AS), descending slope (DS), mean transit time (MTT), and area under the curve (AUC) (**Figure 2**).

### Statistical analysis

The statistical softwares used in this study were SPSS 25.0 (SPSS, Inc, Chicago, IL) and R software (RStudio, Inc., Boston).

A two-sample t-test was used when the measurement data were normally distributed and the variance was uniform. The Mann–Whitney test was used when the measurement data were displayed non-normal distribution. Either Fisher’s exact probability method or the Mann–Whitney test was used to compare count data. Measurement data conforming to normal distribution were expressed as means ± standard deviations (X ± S), those not conforming to normal distribution were expressed as medians (25th, 75th), and count data were expressed as N (%). P<0.05 was considered statistically significant.

## Results

A total of 26 (USMB+NAC: 10, NAC: 16) patients with breast cancer were included in the study. The histological type of all patients was confirmed to be invasive ductal carcinoma by pathology. The general characteristics of the two groups showed no significant differences regarding age, menopausal, pregnancy, and family histories, and other indicators (*P*>0.05) (**Table 1**).

**Table 1:** Baseline characteristics of patients.

	Total (N=26)	UTMB+NAC (N=10)	NAC (N=16)	*P-*value
Age, years	47.460 ± 10.663	42.800 ± 11.263	50.380 ± 9.479	0.077
Menstrual history				0.315
NO	15 (57.69)	7 (70.00)	8 (50.00)	
YES	11 (42.31)	3 (30.00)	8 (50.00)	
Pregnancy history				0.102
NO	4 (15.39)	3 (30.00)	1 (6.25)	
YES	22 (84.61)	7 (70.00)	15 (93.75)	
Family history				0.420
NO	25 (96.15)	10 (100.00)	15 (93.75)	
YES	1 (3.85)	0 (0.00)	1 (6.25)	
Initial Size	35.50 (27.00-48.75)	38.00 (27.00-46.75)	32.50 (29.50-45.00)	0.907
TNM Stage				0.628
2A	1 (3.85)	0 (0.00)	1 (6.25)	
2B	5 (19.23)	3 (30.00)	2 (12.50)	
3A	12 (46.15)	4 (40.00)	8 (50.00)	
3C	8 (30.77)	3 (30.00)	5 (31.25)	
Histological grade				0.395
I	2 (7.69)	0 (0.00)	2 (12.50)	
II	10 (38.46)	5 (50.00)	5 (31.25)	
III	14 (53.85)	5 (50.00)	9 (56.25)	
Subtype				0.664
Luminal B Her2 negative	18 (69.20)	6 (60.00)	12 (75.00)	
Triple negative	8 (30.80)	4 (40.00)	4 (25.00)	
Chemotherapy times	6 (6,6)	6 (4,6)	6 (6,6)	0.515

Data are reported as X ± S, Median (25th,75th), or N (%).

### Safety evaluation

Clinical parameters, including vital signs, electrocardiograms, and blood chemistry, were used to evaluate the toxicity of our treatment. Overall, all data indicated that USMB combined with NAC did not cause any unexpected bias or additional toxicity compared with NAC alone.

**Figure 3:**
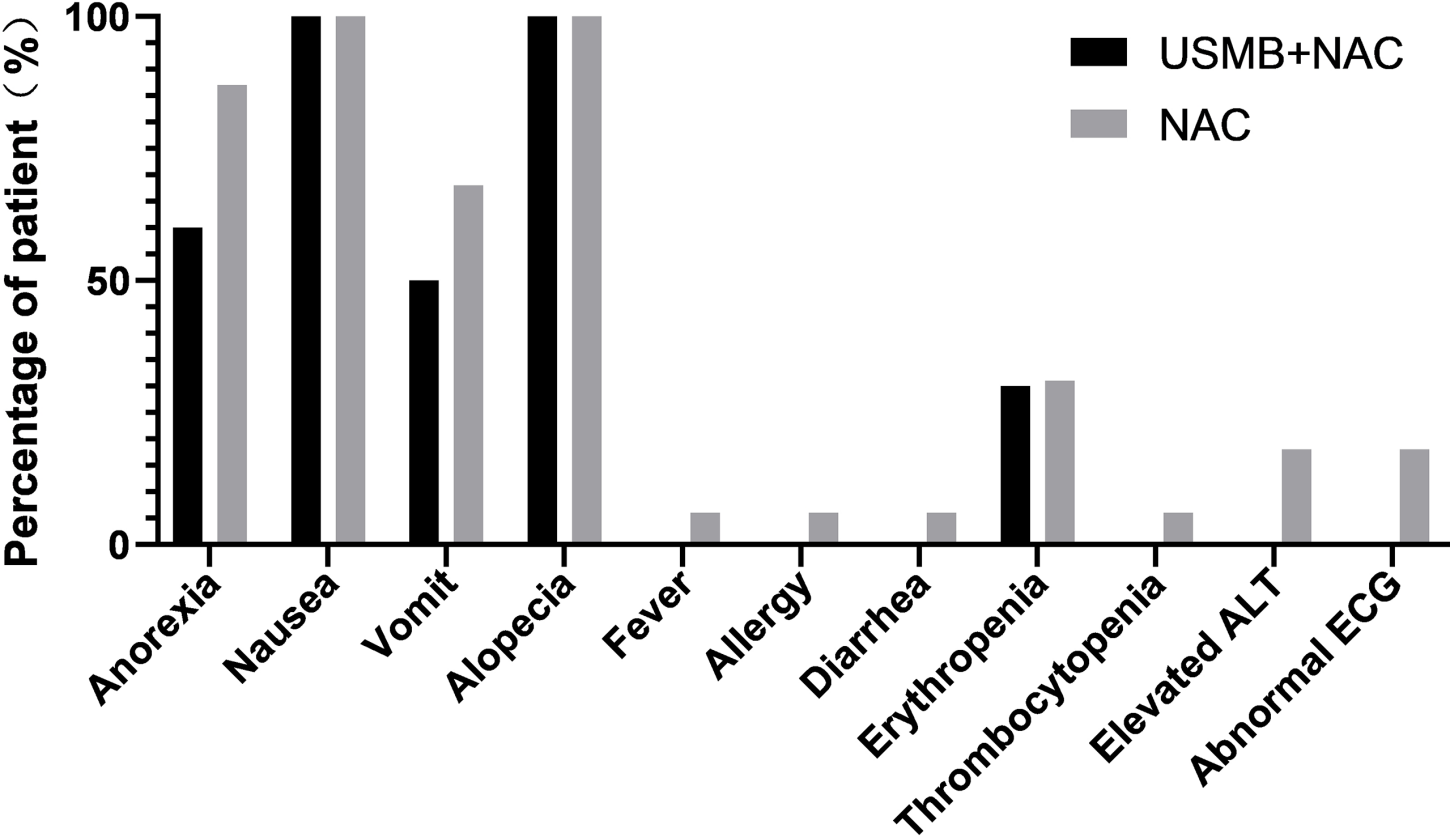
Percentage of patients experiencing adverse events.

There were no serious adverse events and no treatment-related deaths among patients treated with ultrasound. Adverse events included decreased appetite (n=6), nausea (n=10), alopecia (n=10), vomiting (n=5), and erythrocytopenia (n=3), which were possibly related to ultrasound therapy. Because all reported toxicities were expected side effects of NAC, they were assessed as being associated with NAC. Similar results were observed in patients who received NAC alone (**Figure 3**), indicating that USMB combined with NAC did not produce additional toxic reactions.

### Clinical benefit and response assessment

**Figure 4:**
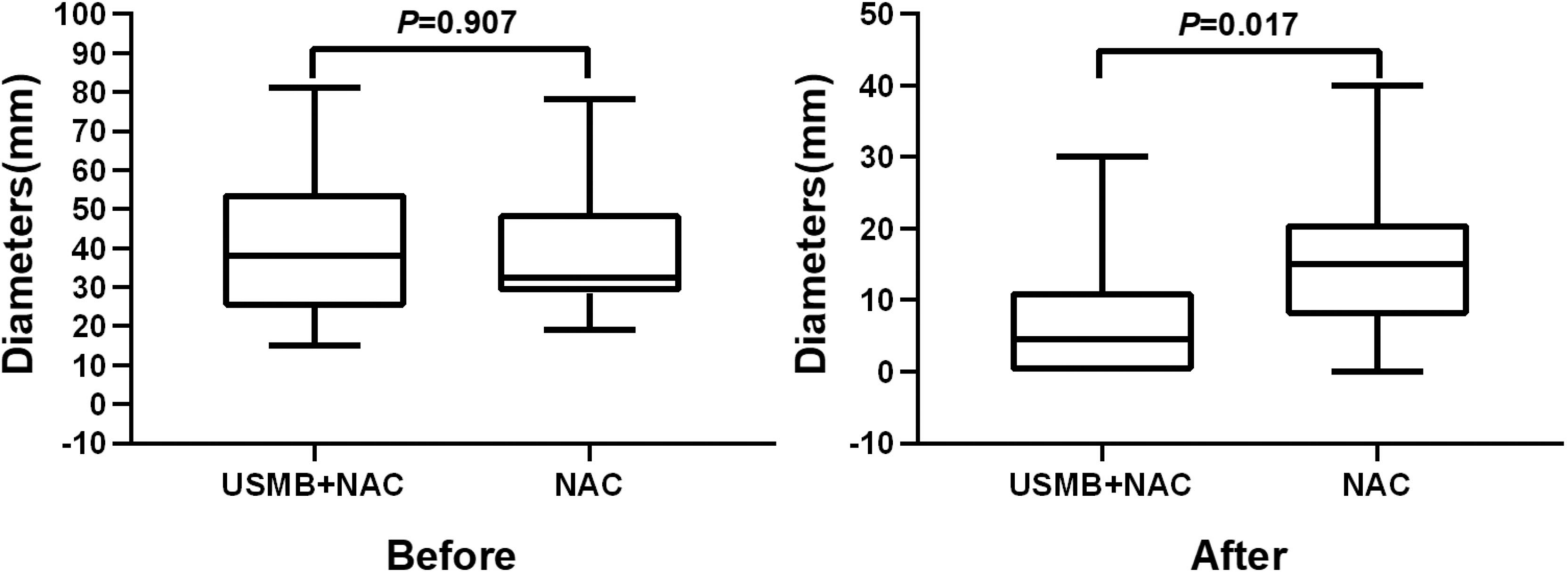
Comparison of the maximum diameter of tumors between the two groups before and after neoadjuvant chemotherapy.

Before NAC, the maximum tumor diameter in the USMB+NAC group (38.00, 27.00–46.75)) was not significantly different from that in the NAC group (32.50 (29.50–45.00)) (*P*=0.907). After the completion of NAC, the maximum diameter of the tumor in the USMB+NAC group (4.50, 0.00–9.25)) was significantly smaller than that in the NAC group (15.00, 9.25–20.25); *P*=0.017 (**Figure 4**).

There was no statistically significant difference in RECIST between the USMB + NAC and NAC groups; *P*=0.590 (**Table 2**).

**Table 2:** Comparison of the response evaluation criteria in solid tumors between USMB+NAC and NAC groups.

	USMB+NAC (n=10)	NAC (n=16)	*P*-value
RECIST			0.590
Complete response	3 (30.00)	2 (12.50)	
Partial response	6 (60.00)	10 (62.50)	
Stable disease	1 (10.00)	3 (18.75)	
Progressive disease	0 (0.00)	1 (6.25)	

Data are reported as N (%).

In the USMB+NAC group, nine patients achieved complete response (CR) and partial response (PR), and one patient achieved stable disease (SD). The overall response rate was 90% and the clinical benefit rate was 100%. In the NAC group, 12 patients achieved CR and PR, three achieved SD, and one achieved PD. The overall response rate was 75%, and the clinical benefit rate was 93.75%; however, the differences were not statistically significant (*P*=0.617, *P*=1.000, respectively).

With regard to postoperative pathological assessment, when MP grading was used to evaluate the complete response rate, 4 cases in the USMB+NAC group reached MP 5, and the pCR rate (40.00%) was significantly higher than that in the NAC group (6.25%), but the difference was not statistically significant (*P*=0.055).

When RCB grading was used to evaluate the complete response rate, four patients in the USMB + NAC group reached RCB grade 0, whereas one patient with MP grade 5 in the NAC group failed to achieve RCB grade 0 due to residual ductal carcinoma in situ, which could not be assessed as pCR. The pCR rate in the USMB+NAC group (40.00%) was significantly higher than that in the NAC group (0.00%); *P*=0.014 (**Table 3**).

**Table 3:** Comparison of pCR rate between USMB+NAC and NAC group.

	USMB+NAC(N=10)	NAC(N=16)	*P*-value
MP			0.055
1-4	6 (60.00)	15 (93.75)	
5	4 (40.00)	1 (6.25)	
RCB			0.014
1-3	6 (60.00)	16 (100.00)	
0	4 (40.00)	0 (0.00)	

Data are represented as N (%).

### Tumor perfusion

**Figure 5:**
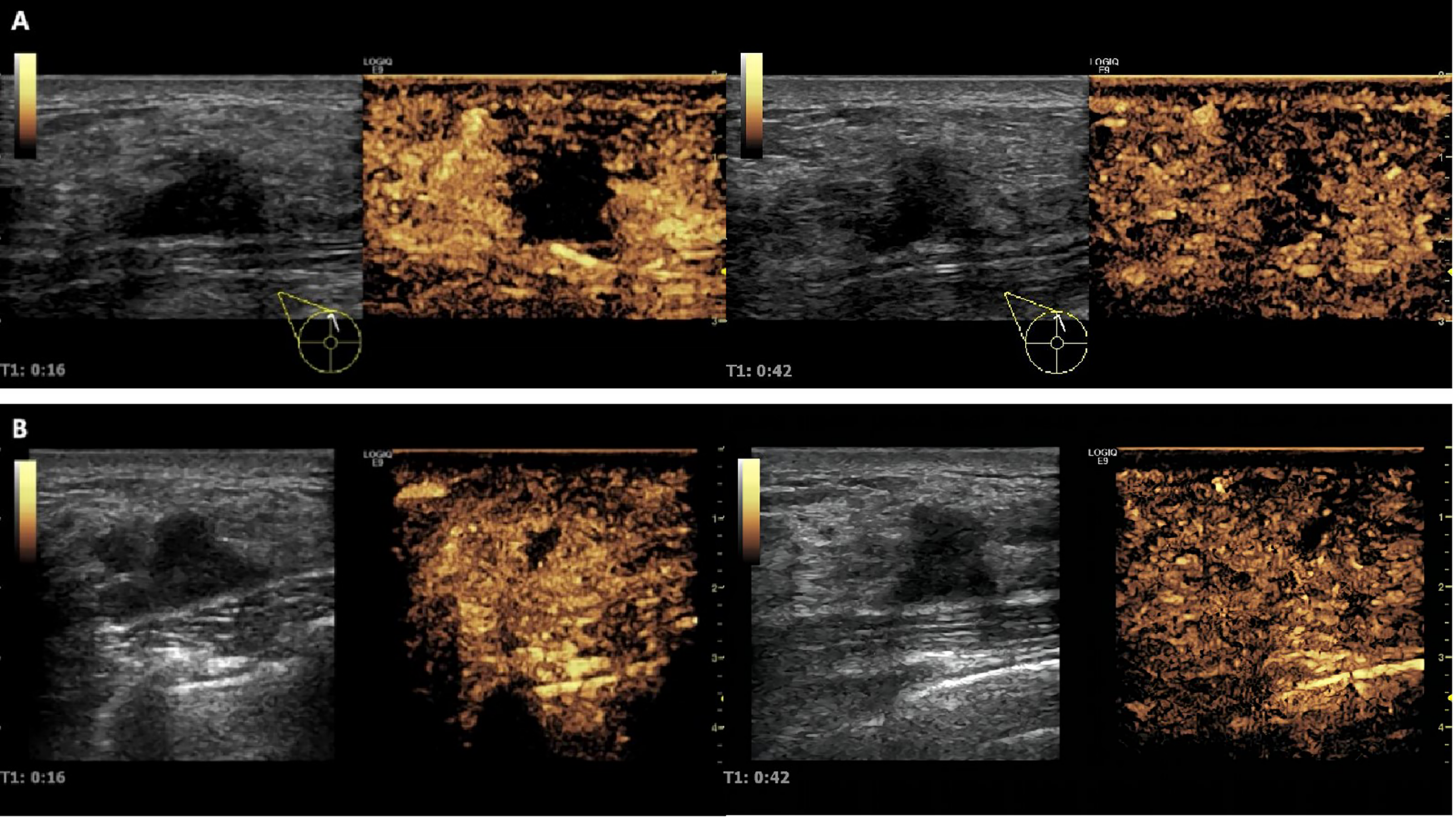
Changes in tumor blood perfusion revealed by CEUS before and after ultrasonic therapy. **(A)** Before ultrasound treatment, CEUS showed a large perfusion defect area in the lesion; **(B)** After ultrasound treatment, CEUS showed that the intrafocal perfusion defect area was significantly reduced, and tumor blood perfusion increased significantly.

**Figure 6:**
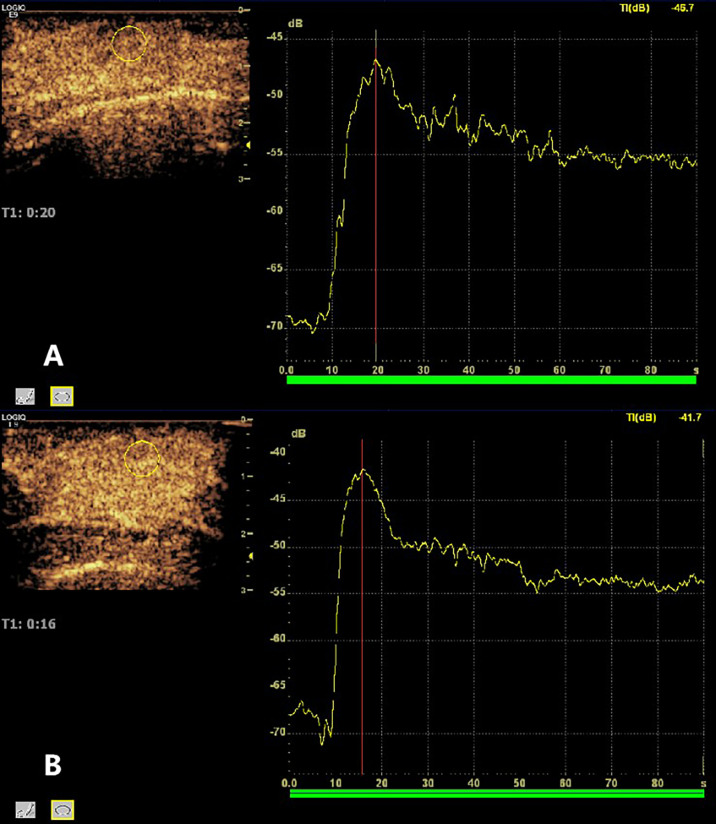
Changes in the CEUS TIC curve before and after ultrasonic therapy. **(A)** Before ultrasound treatment, peak intensity = -45.7dB, mean transit time = 74.00 s, area under curve =1560.317; **(B)** After ultrasound treatment, peak intensity = -41.7dB, mean transit time = 85.60s, and area under the curve = 1746.542.

In the USMB + NAC group, 45 ultrasonic treatments were performed. Quantitative analysis of CEUS images of breast cancer lesions before and after ultrasound treatment revealed that the velocity, intensity, and time of blood perfusion of breast tumors after ultrasound treatment were altered to varying degrees compared to pre-treatment values (**Figures 5**, **6**). Mass after ultrasound treatment showed higher peak intensity (-41.811 ± 3.893 vs. -45.096 ± 3.421, *P*< 0.001), longer mean transit time (62.736 ± 15.287 vs. 50.473 ± 16.529, *P*<0.001) and larger area under the curve (1119.066 ± 321.367 vs. 913.888 ± 307.240, *P*=0.003). Other quantitative parameters of CEUS before and after ultrasound treatment (time to peak, ascending slope, and descending slope) did not exhibit statistically significant differences (**Table 4**).

**Table 4:** Comparison of quantitative parameters of CEUS TIC curve before and after ultrasound therapy.

	Before	After	*P* Value
PI	-45.096 ± 3.421	-41.811 ± 3.893	<0.001
TTP	21.500 (18.700–23.900)	22.000 (19.000–22.000)	0.373
AS	1.530 (1.100–2.100)	1.990 (1.400–2.450)	0.042
DS	0.210 (0.150–0.270)	0.180(0.160–0.250)	0.536
MTT	50.473 ± 16.529	62.736 ± 15.287	<0.001
AUC	913.888 ± 307.240	1119.066 ± 321.367	0.003

Data are represented as X ± S or median (25th,75th). (AS, ascending slope; AUC, area under the curve; DS, descending slope; MTT, mean transit time; PI, peak intensity; TTP, time to peak).

## Discussion

To our knowledge, few clinical trials have reported the use of diagnostic USMB in the treatment of breast cancer, and most previous studies were conducted *in vitro* or in animals. Therefore, the effect of diagnostic USMB in human breast cancer remains unclear. In this study, we collected clinical and imaging data and final pathological findings from all 10 HER2-negative patients to evaluate the efficacy and safety of diagnostic ultrasound combined with contrast agent microbubbles and chemotherapy agents in patients with breast cancer.

### Adverse events

In the current study, we listed all adverse events experienced by patients, which also included those due to actual malignancies or personal experiences. There were no serious adverse events (SAE) or treatment-related deaths among the patients treated with ultrasound. Nausea (100% vs. 100%) and alopecia (100% vs. 100%) occurred in the majority of patients in both groups after NAC. The types of adverse events in the USMB + NAC group were similar to those in the NAC group. Since all reported toxic reactions were expected side effects of NAC, we do not think that these events were related to ultrasound treatment. The incidence of adverse events in the USMB+NAC group was similar to or lower than those in the NAC group, and there were no adverse events such as abnormal liver function, fever, allergy or diarrhea in the ultrasound treatment group that occurred in the NAC group. Generally, all data indicated that USMB combined with NAC did not cause any unexpected bias or additional toxicity compared with NAC alone.

### The response assessment

In this study, tumor diameter is both groups declined, the maximum tumor diameter of USMB+NAC group was smaller than that of the NAC group; the overall response and clinical benefit rates of the USMB+NAC group were higher than those of the NAC group. This may be related to ultrasonic treatment which can increase the local tumor tissue deposition, and inhibit tumor growth (6, 11, 14), However, there was no statistically significant difference between the two groups in the evaluation of response evaluation criteria in solid tumors, which was believe to be related to the small sample size.

In the postoperative pathological assessment, when the MP grading system was used, the pCR rate in the USMB+NAC group was higher than that in the NAC group, but the difference was not statistically significant. Although the MP system is widely used in China, the system only assesses the residual breast cancer cell abundance of primary breast lesions, without evaluation of axillary lymph nodes. When the density of tumor cells is unevenly distributed after chemotherapy, application of the classification system is difficult, owing to the limitations of using the MP assessment system, which is rarely used in international clinical trials (19). The RCB grading system is reproducible and suitable for pathological evaluation of different subtypes of breast cancer after treatment (18). In this study, the pCR rate of the USMB + NAC group evaluated using RCB was significantly higher than that of the NAC group. One patient in the NAC group was evaluated to achieve pCR in MP grading; however, pCR was not achieved in the RCB grading evaluation because of a small amount of residual intraductal carcinoma in situ. Therefore, the pCR rate of the NAC group decreased after RCB grading evaluation, whereas the pCR rate of the USMB+NAC group remained consistent with the MP grading. This analysis was considered to be related to the increased permeability of microvascular endothelial cell membranes in the tumor area caused by ultrasonic therapy, the uptake of more chemotherapy drugs by cells, increased sensitivity of cells to chemotherapy drugs, and more thorough killing of tumor cells (15, 20). Studies have shown a strong correlation between pathological reactions and prognosis after neoadjuvant therapy; patients who achieve pathological complete response (pCR) have a better prognosis after neoadjuvant therapy than patients with residual lesions (3). Therefore, the results of this study suggest that diagnostic ultrasound combined with contrast agent microbubbles can enhance the efficacy of NAC for breast cancer, reduce residual tumor cells in the primary tumor, improve the pCR rate of NAC for breast cancer patients, and help patients achieve a better prognosis.

### Tumor perfusion

We used CEUS to observe the changes in blood perfusion in breast tumors before and after ultrasound treatment. The results of this study showed that after ultrasound treatment, PI, MTT, and AUC increased compared to those before ultrasound treatment, and the differences were statistically significant. The PI is the highest point of the TIC curve, indicating the maximum peak intensity of contrast agent perfusion in the lesion and reflecting the blood volume in the ROI. The AUC is a comprehensive evaluation of velocity, flow rate, and time, reflecting the total blood volume in the ROI within a certain time. After ultrasound treatment, PI and AUC increased compared to those before treatment, suggesting that ultrasound treatment can increase the total blood volume in the ROI within a specific time, which is consistent with the results of Li et al. (21). MTT is the average transit time, which reflects the retention time of the contrast agent microbubbles in the ROI. After ultrasonic treatment, MTT increased significantly compared to that before treatment, suggesting that the retention time of chemotherapy drugs in local tumor tissues increased, which was different from the results of Li et al. (21). This may be due to the different research objects of ultrasonic therapy (breast cancer patients vs. tumor-bearing mice) and different treatment parameter settings (PRF: 20 Hz vs. PRF: 1 KHz), different parameter settings, and different microbubble doses leading to different biological effects of ultrasonic therapy on tumor cells. There are differences in the influence of tumor microvessels (22). Previous *in vivo* studies have demonstrated that microvessel hemorrhages and alterations of endothelial permeability can be produced in tissues containing microbubble-based ultrasound contrast agents when those tissues are exposed to MHz-frequency pulsed ultrasound of sufficient pressure amplitudes. The experimental results of Hwang et al. suggested that microbubble contrast agent could reduce the threshold ultrasonic intensity. Endothelial cell damage and erythrocyte extravasation could be observed in rabbit auricular vessels under 1Mhz ultrasonic irradiation (23). Since the diameter of microbubbles (2-4μm) is smaller than that of red blood cells (6-8μm), microbubbles and red blood cells may extravasate under low-intensity ultrasound treatment which reduces the speed of microbubble exiting the ROI (DS, 0.210 (0.150–0.270) VS 0.180(0.160–0.250), *p*=0.536) and thus prolongs the residence time of microbubbles.

The time to peak (TTP) in the CEUS TIC curve reflected the time when contrast agent perfusion reached the peak, and the ascending slope (AS) and descending slope (DS) reflected the changes in blood volume in contrast agent microbubbles entering and exiting the ROI with time, respectively. In this study, there were no statistically significant differences between TTP, AS, and DS before and after treatment, which may be affected by the injection time and speed of the contrast agent.

### Limitations

Although these results show great promise, we cannot make global assertions regarding the efficacy of USMB-enhanced NAC for breast cancer based on this study. To further understand and validate these results, it is important to perform mechanistic experimental studies and investigate larger patient cohorts in prospective randomized controlled trials.

Our study did not investigate the therapeutic effects of NAC for breast cancer using different ultrasound irradiation modes. The ultrasound emission conditions were severely limited by the clinical diagnostic scanner. In previous studies, high MI contrast-enhanced Doppler ultrasound (CDUS) did not influence morphological and functional vascular characteristics of breast cancer in patients. And complete clinical tumor response after neoadjuvant chemotherapy was lower in high MI CDUS-treated compared to untreated patients (24). Some researchers have suggested that a low mechanical index provided a better therapeutic effect than a high mechanical index (21, 25). Future work should aim to determine the ultrasound conditions that induce the highest therapeutic effect to facilitate implementation of such conditions in clinical practice.

Due to the difficulty of puncture sampling before and after each ultrasound treatment, this study failed to detect the local tumor drug concentration before and after ultrasound treatment in real time to explore the influence of the increase of blood perfusion on the local drug concentration. Future work should aim to explore appropriate methods to obtain the drug concentration of local tumor tissue to explore the effect of ultrasound treatment on local drug concentration.

## Conclusions

In the USMB cohort, CEUS showed that the blood flow in the lesion increased compared with that before ultrasonic treatment, more patients achieved pCR and obtained a better prognosis. USMB combined with chemotherapy showed no additional toxicity. These novel results show great promise for USMB-enhanced NAC for HER2-negative breast cancer.

## Data availability statement

The original contributions presented in the study are included in the article/supplementary material. Further inquiries can be directed to the corresponding authors.

## Ethics statement

The studies involving human participants were reviewed and approved by the Ethics Committee of the Second People’s Hospital of Shenzhen. The patients/participants provided their written informed consent to participate in this study.

## Author contributions

XG and BZ designed the study. PZ, QL, CJ, and LX acquired the data. QL, CJ, and JL contributed to manuscript preparation and data analysis. BZ and XG revised the manuscript. All authors contributed to the article and approved the submitted version.

## Funding

This study was supported by a grant from the Shenzhen Science and Technology Innovation Foundation (grant number: JCYJ20170413161913429), Shenzhen Key Medical Discipline Construction Fund (SZXK052), Sanming Project of Medicine in Shenzhen (SZSM201612027) and Clinical research project of shenzhen Second people’shospital (20203357001).

## Acknowledgments

We would like to thank Editage (www.editage.cn) for English language editing.

## Conflict of interest

The authors declare that the research was conducted in the absence of any commercial or financial relationships that could be construed as a potential conflict of interest.

## Publisher’s note

All claims expressed in this article are solely those of the authors and do not necessarily represent those of their affiliated organizations, or those of the publisher, the editors and the reviewers. Any product that may be evaluated in this article, or claim that may be made by its manufacturer, is not guaranteed or endorsed by the publisher.

## Abbreviations

AS, ascending slope; AUC, area under the curve; CEUS, contrast-enhanced ultrasound; CR, complete response; DS, descending slope; HR, Hormone receptor; NAC, neoadjuvant chemotherapy; MTT, mean transit time; MP, Miller–Payne; pCR, pathologic complete response; PI, peak intensity; PR, partial response; RCB, residual cancer burden; ROI, region of interest; SD, stable disease; TAC, taxane (docetaxel), anthracycline (epirubicin or doxorubicin liposomes), and cyclophosphamide; TTP, time to peak; USMB, ultrasound combined with microbubbles.
